# Coach Mid-Season Replacement and Team Performance in Professional Soccer

**DOI:** 10.2478/v10078-011-0028-7

**Published:** 2011-07-04

**Authors:** Carlos Lago-Peñas

**Affiliations:** 1 Faculty of Education and Sports Sciences, University of Vigo, Pontevedra, Spain

**Keywords:** coach change, soccer, team performance, short term

## Abstract

The coaching carousel or turnover is an extreme but frequently occurring phenomenon in soccer. Among the reasons for firing a coach, the most common is the existence of a shock-effect: a new coach would be able to motivate the players better and therefore to improve results. Using data from the Spanish Soccer League during the seasons from 1997–1998 to 2006–2007, this paper investigates the relationship between team performance and coach change over time. The empirical analysis shows that the shock effect of a turnover has a positive impact on team performance in the short term. Results reveal no impact of coach turnover in the long term. The favourable short-term impact on team performance of a coach turnover is followed by continued gradual worsening of results. The turnover effect is nonexistent when the comparison between the new coach and the old coach is done over 10, 15 or 20 matches before and after termination.

## Introduction

For more than 50 years, researchers have attempted to determine whether sports coaches do matter and have an impact on team performance. One way of addressing this question is to focus the relationship between coach turnover and team performance. Although initial research was in fact management research (Grusky, 1963; Gamson and Scotch, 1964; Eitzen and Yetman, 1972; Allen et al., 1979; Brown, 1982; Pfeffer and Davis-Blake, 1986), more recent studies approached the issue from a sport management perspective (Theberge and Loy, 1976; Fabianc, 1984; 1994; Curtis et al., 1986; McTeer and White, 1986; Salomo and Teichmann, 2000; Bennet et al., 2003; Balduck and Buelens, 2007). Overall, most researchers agree that bad results are the major determinant of a turnover (Cannella and Rowe, 1995; Audas et al., 2002; Salomo and Teichmann, 2000; Bruinshoofd and Ter Weel, 2003).

Kesner and Sebora (1994) argued that three sociology succession theories that were introduced by Gamson and Scotch (1964) have dominated successive research. First, the common-sense theory acknowledges the positive influence of manager turnover on organizational effectiveness. Performance improves following succession. For a team experiencing a run of bad results, a change of coach may provide the stimulus essential to break the sequence. In other words, the change itself may have a beneficial psychological and motivational effect on players, even if objectively the attributes of the incoming coach are no different from those of his predecessor. Second, the vicious-circle theory accepts the reciprocal effect of a resignation. Turnover, frequently caused by poor performance, disrupts internal relationships in an organization. The resulting destabilizing force leads to a further decline in performance. The third explanation, the ritual scapegoating theory (Kesner and Sebora, 1994) assumes that a succession has no impact on team performance. Dismissing a coach is a convenient mean of placating frustrated stakeholders.

Some studies found evidence to support the ritual scapegoating theory (Eitzen and Yetman, 1972; Cannella and Rowe, 1995), whereas other studies argued the common-sense theory was more appropriate (Pfeffer and Davis-Blake, 1986; Fabianic, 1994; McTeer and White 1995; Van Dalen, 1997; Bennet et al., 2003). Few studies empirically supported the vicious-circle theory (Brown, 1982).

Several authors discussed the influence of regression to the mean (Salomo and Teichmann, 2000; Audas et al., 2002; Nevill et al., 2004; Rowe et al., 2005) or the slump-ending effect (Gamson and Scotch, 1964). In a stochastic environment, unusually low or high scores will be statistically followed by scores that tend to be closer to the mean. After controlling for regression to the mean, most studies (Curtis et al., 1986; Cannella and Rowe, 1995; Bruinshoofd and Ter Weel, 2003; Koning, 2003) found no succession effect. Audas et al. (1997) suggest that there is a natural tendency for results to improve (on average) after a poor run of results, simply because no team carries on losing forever.

Comparison of the performance of a resignation control group – where no coach turnover had taken place – also revealed paradoxical results. Eitzen and Tetman (1972) as well as Brown (1982) found no differences in team performance between the resignation group and the control group, whereas Audas et al. (1997) found that English soccer clubs that dismissed their coaches performed worse immediately after the turnover than those that retained their coaches. The results of Bruinshoofd and Ter Eeel (2003) revealed that coach turnover did not lead to an improvement in team performance. Moreover, the control group more rapidly recovered to the mean performance compared with the resignation group.

Within the framework of coach turnover, several studies focused on variables such as game location, team quality, coaching experience or coaching ability. Implementing both team quality and home team advantage, Koning (2003) found that team performance did not always improve when a coach was fired within the season. Cannella and Rowe (1995) proved that coaching ability most strongly affected performance when a turnover occured in a high rivalry context, whereas ability had no effect on team performance under conditions of low rivalry. Coaching experience had no impact on team performance after a succession (Balduck and Buelens, 2007).

The ambiguity of the findings creates an interesting challenge. Although some studies concentrated on between-season succession (Allen et al., 1979; Scully, 1995; Rowe et al., 2005), in this paper the thesis that within-season successions are most appropriate for revealing the real effect of a coach resignation is accepted (Salomo and Teichmann, 2000; Balduck and Buelens, 2007). The aims of the current study are: (i) to examine whether firing a coach leads to an improvement of the performance of the team; (ii) to asses the effectiveness of coach turnover over time. While considerable attention has been given to examine the reasons of coach changes in soccer, few studies have analyzed the relationship between coach turnover and team performance over time. Is the effect of a coach change different in the short and long term? Does the *winning effect* of a new coach remain constant or disappear over time? According to the arguments of the common-sense theory, it should be assumed that the slope of this relationship is linear, which means that the favourable impact of a coach change on team performance remains constant over time. But there are no studies that support empirically this assumption or the opposite.

The hypothesis of the current study is that a coach termination has a favourable short-term impact on team performance. According to the common-sense theory, the removal of an unsuccessful coach may galvanise players into a greater effect by raising morale, thereby creating a short term improvement. However, when the psychological effect of a new coach disappears, the turnover effect is non-existent in the long term. Table 1 provides a schematic summary of the logic of the four alternatives this paper considers.

## Methods

### Participants

Data consist of male soccer teams that played in the highest national division and the second division in Spain during the seasons from 1997–1998 to 2006–2007. Table 2 presents the number of coach changes analyzed in the sample. Data were taken from the official statistic of the organizers of the competition in Spain: the Real Federación Española de Fútbol (www.rfef.es) and the Liga de Fútbol Profesional (www.lfp.es).

Data have been noted by two research groups and cross-checked for inter-system accuracy. The percentage agreements equal to 100% was obtained. The season average coach turnover and the standard deviation are 27.6 and 4.27 respectively.

### Procedures

The performance measure is the percentage of points gained by teams in the matches 1, 2, 3, 5, 10, 15 or 20 prior and following to date of turnover. The summary match results statistic is the `win ratio′, calculated by awarding 3 points for a win, 1 for a draw and 0 for a defeat, and the averaging over the relevant spell of matches. No points are awarded when the team loses the game. That is:

[1] Win ratio=Number of points gained / Total number of points

The advantages of this method are twofold. First, a performance measure that can decline when performance stagnates can be obtained. Second, abrupt performance declines or increases are smoothed out. Defining short and long term rests on an arbitrary decision: we must keep in mind some threshold to separate them. Instead of having a dichotomous variable with only two periods of time, in this paper several moments across the season were selected to compare how team performance changed.

Effectiveness of a coach change denotes than team performance under the authority of a new coach improves significantly compared with that under the previous coach. Therefore, the comparison between the mean team performance levels with the old and a new coach is done over 1, 2, 3, 5, 10, 15 and 20 matches before and after the date of resignation.

### Statistical Analysis

A mean comparison test is conducted to evaluate the effect of a coach turnover on mean team performance levels over time. To prove the findings noted above, different linear regression analysis are estimated. As had been said, the dependent variable is the percentage of points gained by teams in the matches 1, 2, 3, 5, 10, 15 or 20 prior and following to date of turnover. When interpreting the statistical results, positive or negative coefficients indicate a greater or lower propensity to improve team performance, respectively. Two independent variables are included in the models. The first variable identify whether the observed coach is the dismissed coach or his successor: COACH. It is a dichotomous variable: 1 = the observed coach is the new coach, 0 = the observed coach is the old coach. The second variable is the number of matches played by teams prior and following to date of turnover: MATCH.

Two linear regression models are used to test the previous hypotheses: (i) an additive model with the variables *coach* and *match* as regressors; (ii) an interactive model, in which a multiplicative combination of the *new coach* and the *number of matches* played by teams prior and following to date of turnover is added to the previous model. The models are as follows:

[2] (NNC−NOC)=β1+β2⋅COACHi+β3⋅MATCHi+εii

[3] $$(N_{\mathrm{NC}}-N_{\mathrm{OC}})=\beta_{1}+\beta_{2}\cdot {\mathrm{COACH}}_{i}+\beta_{3}\cdot {\mathrm{MATCH}}_{i}+\beta_{4}\cdot ({\mathrm{COACH}}_{i}\cdot {\mathrm{MATCH}}_{i})+\varepsilon_{i}$$

## Results

**Figure 1:**
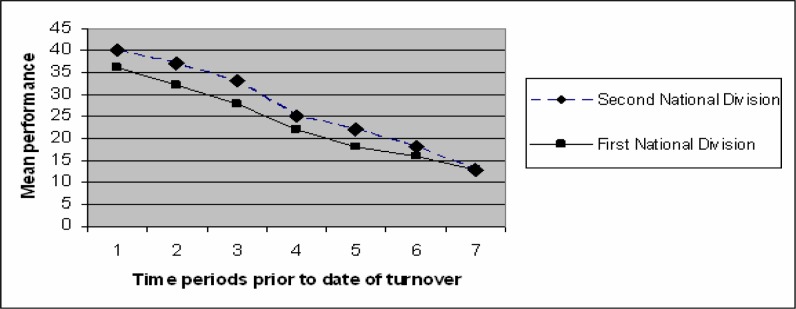
Mean team performance levels of the seven periods prior to turnover for the first and second national divisions in Spanish soccer.

Figure 1 presents information on the match results achieved by coaches during the games played immediately before termination. As it may be seen, team performance is getting poorer by the day, but over the five previous matches to a coach turnover, team performance sharply declines in the two divisions.The second national division has higher performance levels compared with the first national division.

Table 3 reports the average win ratios for selected numbers of matches preceding and following a coach turnover. These figures appear to substantiate the view that coach turnover creates a short-term improvement in performance. The respectively average win ratios in the 1, 1–2, 1–3 and 1–5 matches before termination were 12.9, 17.4, 20.1, 23.8 and after termination 42.18, 43.12, 42.69 and 41.76, respectively, indicating an immediate reversal of the pre-termination decline (*p* <.01). However, these results are followed by continued gradual decreasing in the difference between the team performance obtained by the dismissed coach and his successor. The respectively difference in the average win ratios in the 1–10, 1–15 and 1–20 matches between the new and the old coach was 12.03, 9.65 and 7.38, respectively. Only in the last case the difference is not statistically significant. The favourable short-term impact on team performance of a coach turnover is followed by continued gradual worsening in the long term.

### 

#### The impact of coach change on team performance

In the first regression model, the impact of coach change on team performance is analyzed. The variable *coach* has the expected coefficient: team performance under the authority of the new coach improves by 20% compared with that under the old coach (*p* <.01). The percentage of points gained by the old coach in the analyzed matches is 18.35%. Moreover, every day of the competition increases in 0.75% the percentage of points gained by teams. This model explains about 19% of the variance of team performance. These results demonstrate the positive influence of coach change on team performance and supports empirically the arguments of the common-sense theory. For a team experiencing a run of bad results, a change of coach may provide the stimulus required to break the sequence.

#### The impact of coach change on team performance over time

In the second regression model, the impact of coach change on team performance over time is analyzed. The inclusion of the interaction between *coach* and *day* increases the impact of the new coach on team performance from 20.24 to 27.18% (p < 0.01). The interaction has the anticipated sign: the greater the number of matches under the authority of the new coach, the worse the team performance. The favourable short-term impact on team performance of a coach turnover is followed by continued gradual worsening in the long term. Each day under the authority of the new coach decreases team performance by 1.21%. The percentage of points gained by the old coach in the first day of the competition is 14.89%. Moreover, every day of the competition increases by 1.36% the percentage of points gained by teams. This model explains about 20% of the variance of team performance.

**Figure 2:**
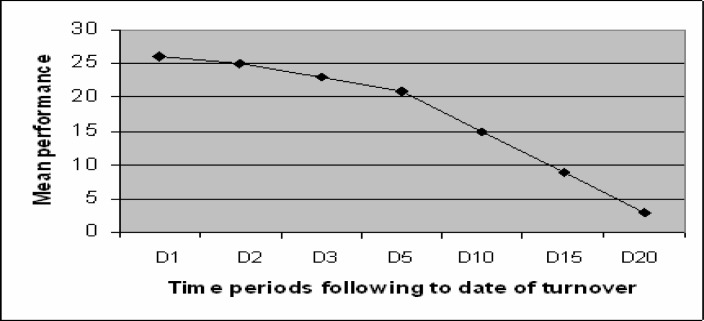
Evolution over time of the winning effect of the new coach

According to the results of the second regression model, in Figure 2 the evolution of the *winning effect* of the new coach over time is presented. The line is the difference between the average team performance obtained by the dismissed coach and his successor. As it can be seen, the favourable short-term impact on team performance of a coach turnover is followed by continued gradual worsening in the long term.

**Figure 3:**
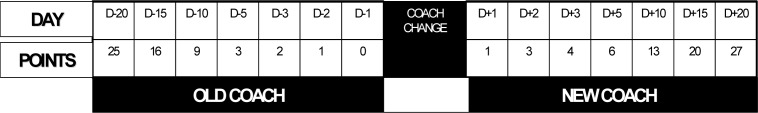
Comparison between the points gained by the old coach and the new coach in several periods prior and following to date of turnover

Finally, to illustrate the findings, estimates of actual and simulated punctuations for a team under the authority of the new and old coach over time are displayed. What punctuation would be predicted for new coaches over time? It is similar for new and old coaches? In Figure 3, simulated punctuations for dismissed coaches and their successors in several periods prior and following to date of turnover are presented.

## Discussion

The coaching carousel or turnover is an extreme, but frequently occurring phenomenon in soccer. Among the reasons for firing a coach, the most common is the existence of a shock-effect: a new coach would be able to motivate better the players and therefore to improve results. This study investigates the relationship between team performance and coach change over time.

During seven consecutive periods prior to a coach turnover, team performance sharply declined after which the observed teams dismissed their coach. Mean team performance increased after the turnover. Team performance under the authority of the new coach improves significantly compared with that under the old coach in the short term but this impact is negligible in the long term. The turnover effect is non-existent when the comparison between the new coach and the old coach is done over 10, 15 or 20 matches before and after termination. The results point out that the favourable short-term impact on team performance after a coach change should be explained by the psychological and motivational effect on players provided by the coach turnover (Pfeffer and Davis-Blake, 1986; Fabianic, 1994; McTeer and White, 1995: Bennet et al., 2003). Conventional wisdom suggests that it takes time for new coaches to accumulate organization-specific knowledge (Salomo and Teichmann, 2000; Audas et al., 2002). Balduck and Buelens (2007) argued that a period of approximately one month - that is four or five matches - might be too short for new coaches to reconstruct the team according to the way they want to play the game, so coaching ability until match four or five after a coach change should have no impact on team performance. When the psychological effect of the new coach disappears, the ability of the coach to lead the team is the most important variable of team performance.

These results demonstrate the positive influence of coach change on team performance and support empirically the arguments of the *common-sense theory*. For a team experiencing a run of bad results, a change of coach may provide the stimulus required to break the sequence.

The empirical evidence supporting the *ritual scapegoating theory* (Kesner and Sebora, 1994) is not totally compelling for two methodological reasons. First, team performance is not a random variable. Obviously, a certain degree of randomness or unpredictability is inherent in team sports, but this fact does not mean that winning would be a function of chance.

Second, the comparison of the performance between soccer clubs that dismissed their coaches and other teams with an identical (or very similar) pattern of results where no coach turnover had taken place does not satisfy the causal homogeneity assumption (King et al., 1994). There are much more differences between teams apart from the coach, for example, the club budget, the style of play, the quality of the players or the history of the club.

A limitation of this study is that the paper did not consider the effect of home team advantage and team quality. The study also did not control player motivation to perform, nor did the paper control the stage of the season when coach turnover occurred. Both variables may have an effect on team performance. Further research should incorporate these possible determinants.

## Conclusions

Using data from the Spanish Soccer League during the seasons from 1996–1997 to 2006–2007, this paper investigates the relationship between team performance and coach change. During seven consecutive periods prior to a coach turnover, team performance sharply declined, after which many clubs dismissed their coach. Mean team performance increased after turnover. The empirical analysis shows that the shock effect of a turnover has a positive impact on team performance over time. Results reveal no impact of coach turnover in the long term. The favourable short-term impact on team performance of a coach turnover is followed by continued gradual worsening in the half of a term. The turnover effect is non-existent when the comparison between the new coach and the old coach is done over 10, 15 or 20 matches before and after termination.

## Figures and Tables

**Table 1:** Impact of coach change on team performance over time according to the vicious-circle theory, the ritual scapegoating theory, the commom-sense theory and the current study

**Theory**	**Favourable impact of coach change on team performance**	**Slope of the favourable impact over time**
Vicious-circle theory	NO	
Ritual scapegoating theory	NO	
Common-sense theory	YES	=
Current study	YES	> in the short term and = in the long term

**Table 2:** Number of coach changes in the first and the second national division during the seasons from 1997–1998 to 2006–2007 in the Spanish Soccer League

**Season**	**N. of coach changes**	**Percentage %**
2006–2007	24	8.70
2005–2006	27	9.78
2004–2005	26	9.42
2003–2004	32	11.59
2002–2003	27	9.78
2001–2002	26	9.42
2000–2001	22	7.97
1999–2000	24	8.70
1998–1999	34	12.31
1997–1998	34	12.31
**TOTAL**	**276**	**100**

**Table 3:** Win ratios before and after coach termination Standard deviations in parentheses. ^*^p < .01.

	1 match before	1 match after	2 mat. before	2 mat. after	3 mat. before	3 mat. after		

Observations	276		264		240			
Win ratios	12.97 (25.58)	42.18 (42.32)	17.42 (20.12)	43.12 (28.92)	20.05 (16.88)	42.69 (23.45)		
Difference	29.21 (35.57)		25.70 (34.26)		22.64 (28.602)			
*T*	9.39		12.18		12.24			

*P > t*	0.000^*^		0.000^*^		0.000^*^			

	5 mat. before	5 mat. after	10 mat. before	10 mat. after	15 mat. before	15 mat. after	20 mat. before	20 mat. after

Observations	205		118		42		7	
Win ratios	23.77 (13.83)	41.76 (18.76)	30.17 (11.61)	42.20 (14.12)	34.03 (10.29)	43.68 (11.44)	39.05 (9.22)	46.43 (9.45)
Difference	17.99 (21.79)		12.03 (14.54)		9.65 (13.52)		7.38 (13.74)	
*T*	11.83		8.99		5.15		1.42	

*P > t*	0.000^*^		0.000^*^		0.000^*^		0.205	

**Table 4:** The impact of coach change on team performance

***Dependent Variable:*** *Percentage of points gained by teams*	***Models***	
***Independent variables***	**1**	**2**
*Coach*	20.25^*^ (0.87)	27.18^*^ (1.41)
*Match*	0.75^*^ (0.06)	1.36^*^ (0.07)
*Coach x Match*		−1.21^*^ (0.08)
*Constant*	18.35^*^ (0.68)	14.89^*^ (0.78)
*R^2^*	0.19	0.20
*N*	2878	2878

Estimation is by ordinary least squares. Robust standard errors are given in parentheses.

* p < .01.
